# Predictors of Improvement in Concomitant Tricuspid Regurgitation Following Transcatheter Edge-to-Edge Mitral Valve Repair

**DOI:** 10.3390/jcm12196191

**Published:** 2023-09-25

**Authors:** Matthias Gröger, Kai Hirsch, Dominik Felbel, Michael Paukovitsch, Leonhard Moritz Schneider, Sinisa Markovic, Wolfgang Rottbauer, Mirjam Keßler

**Affiliations:** Department of Internal Medicine II, Ulm University Heart Center, 89081 Ulm, Germany; matthias.groeger@uniklinik-ulm.de (M.G.); kai.hirsch@uni-ulm.de (K.H.); dominik.felbel@uniklinik-ulm.de (D.F.); michael.paukovitsch@uniklinik-ulm.de (M.P.); leonhard-moritz.schneider@uniklinik-ulm.de (L.M.S.); sekimehi@adk-gmbh.de (S.M.); sekretariat.rottbauer@uniklinik-ulm.de (W.R.)

**Keywords:** tricuspid regurgitation, mitral regurgitation, mitral valve repair, TEER

## Abstract

Background: Improvement in concomitant tricuspid regurgitation (TR) after mitral valve transcatheter edge-to-edge repair (M-TEER) for mitral regurgitation (MR) occurs frequently; however factors determining the post-procedural course of TR are not well understood. We investigated the parameters associated with TR improvement after M-TEER. Methods and Results: A total of 300 patients were consecutively included in this retrospective analysis. MR and TR severity as well as heart chamber metrics were assessed before the procedure and at follow-up. Device success was achieved in 97.3% of patients. TR decreased in 30.2% of patients. Patients with improved TR were more often female, had more severe TR at baseline, and their right heart dimensions at baseline trended to be smaller. Female sex (odds ratio (OR) 2.997), baseline MR-Grade (OR 3.181) and baseline TR-Grade (OR 2.653) independently predicted TR reduction. More pronounced right heart reverse remodeling was observed in patients with improved TR. TR regression independently predicted lower mortality (hazard ratio (HR) 0.333, 95% confidence interval 0.112–0.996, *p* = 0.049). Conclusions: A reduction in concomitant TR severity after M-TEER occurred mainly in females and in patients with high-grade TR and MR at baseline. TR regression is associated with better survival after M-TEER.

## 1. Introduction

Percutaneous transcatheter edge-to-edge mitral valve repair (M-TEER) is well-established for interventional treatment of moderate-to-severe mitral regurgitation (MR) in symptomatic patients with high surgical risk and favorable anatomy [1]. MR can either occur due to leaflet degeneration (primary MR, PMR) or due to left ventricular or left atrial dilation with impaired motion and tethering of the leaflets (secondary MR, SMR) [1]. Since left heart disease is also the most common cause of tricuspid regurgitation (TR) [2], MR and TR often occur concomitantly, and symptoms of biventricular heart failure can overlap. Moreover, it is known that right ventricular (RV) dysfunction and severe concomitant TR are associated with adverse outcomes in patients undergoing M-TEER [3,4,5,6]. Regression of TR is often achieved by mitral valve repair alone, and is described in one third to up to 50% of patients undergoing the procedure [3,7,8]. Improvement in left ventricular hemodynamics with induction of left ventricular reverse remodeling and consecutive reduction of left atrial and pulmonary artery pressures have been proposed as the underlying mechanism of TR reduction [9,10]. Accordingly, more pronounced TR reduction has been linked to significant reduction in systolic pulmonary artery pressure (sPAP) following M-TEER [8]. While the subsequent reduction in TR is associated with better survival after M-TEER [11], the absence of TR reduction following mitral valve repair is often paralleled by an impaired mid- and long-term outcome, especially in SMR patients [5,11]. Improvement in TR severity is seen more regularly in patients with RV dysfunction at baseline [3]. However, factors predicting RV reverse remodeling and improvement in TR are not yet understood. Kavsur et al. identified atrial fibrillation, residual MR grade ≥ II and tricuspid annular dilation as being preventive of TR regression [12]. In a large study by Geyer et al., patients with TR regression had higher baseline sPAP as well as higher baseline systolic right ventricular function, as assessed by TAPSE (tricuspid annular plane systolic excursion) [5]. Interventional treatment of TR, e.g., by transcatheter edge-to-edge tricuspid valve repair (T-TEER), is a possible therapeutic option for patients with symptomatic high-grade TR [1] and studies evaluating combined M-TEER and T-TEER have already been carried out [13]. However, given the large proportion of patients with TR regression from mitral valve repair alone, it seems feasible that a substantial number of patients can be managed with a “wait-and-observe“ strategy. Understanding the course of TR following M-TEER is crucial in order to select patients who will benefit from early secondary tricuspid valve repair. Our study therefore aimed to characterize patients with improved TR after mitral valve repair and to identify baseline factors predicting TR regression.

## 2. Materials and Methods

A total of 300 patients underwent M-TEER for moderate-to-severe MR at our center from October 2017 to January 2021 and were consecutively included in this retrospective analysis. All patients included in the present study were symptomatic in terms of heart failure (New York Heart Association (NYHA) functional class ≥ II) despite guideline-directed medical therapy. All patients underwent diagnostic work-up prior to M-TEER, as previously described [14].

Echocardiographic characteristics at baseline were available for all study patients. TR at baseline and during follow-up was assessed quantitatively by biplane vena contracta measurement using the 5-grade system [15]. Severity of MR was classified in four degrees according to the latest EACVI/ESC recommendations for MR quantification [16]. LV-EF was measured using the biplane Simpson’s method. Left atrial volume index (LAVI) was measured using transthoracic echocardiography from the apical four-chamber and two-chamber view, and right atrial volume index (RAVI) and RV dimensions were measured from the apical four-chamber view. LV end-systolic (LVESD) and end-diastolic diameter (LVEDD) were measured in the parasternal long-axis view. Post-procedural MR severity was assessed using 2D and 3D transesophageal echocardiography after final device placement and removal of the guide catheter. MR was semi-quantitatively assessed by visual estimation of the MR jet area and by (biplane) determination of the vena contracta of the major MR jet. In addition to MR severity, the mitral valve gradients and area were assessed using a 3D technique before and after clip deployment and after removal of the guide catheter. 

Device success was defined as TEER with reduction in MR of at least one degree and absence of major device- or procedure-related serious adverse events [17].

Analysis was carried out in two groups: patients with TR improvement by at least one grade at any follow-up visit within 12 months after M-TEER vs. patients with no TR improvement within 12 months. Patients who underwent tricuspid valve procedures within 12 months were excluded from this analysis. Patients were analyzed as a combined cohort of PMR and SMR. Echocardiographic follow-up was available for 198 patients. The occurrence of all-cause mortality was analyzed through a 12-month follow-up period. 

Statistical analysis was performed using SPSS 28 software (IBM Corp., Armonk, NY, USA). Categorical variables are expressed as counts and percentages and were compared using chi-square test or McNemar’s test, as appropriate. Continuous parameters are presented as the mean ± standard deviation and were compared using the Kruskal–Wallis test or *t*-test, as appropriate. All-cause mortality was analyzed using log-rank analysis. 

To identify factors independently associated with TR improvement, a multivariate logistic regression was carried out including potentially influential baseline variables (*p* < 0.2). Due to significant correlation with baseline MR grade, and since pre-procedural parameters of influence on TR regression were of interest, absolute MR reduction was excluded from the multivariate logistic regression analysis. To determine predictors of adverse outcome, univariate Cox regression analysis was performed for all potential influential variables (*p* < 0.2). Using the multivariate Cox regression analysis, a backward stepwise algorithm was applied to all potential influential parameters (*p* < 0.2) from the univariate Cox regression analysis. Differences were considered statistically significant when *p* < 0.05. 

## 3. Results

### 3.1. Procedural Outcome of M-TEER

A total of 300 patients were consecutively included in this analysis. Before the M-TEER, 82.3% of patients had MR grade IV, 16.7% had grade III and 0.7% had dynamic grade II. TR grade at baseline was V in 2.0%, IV in 9.4%, III in 34.4%, II in 41.1% and I in 13.0%. Device success was achieved in 97.3% of patients. A total of 92.2% of patients were discharged with MR grade ≤ II. No periprocedural deaths occurred. 

**Figure 1:**
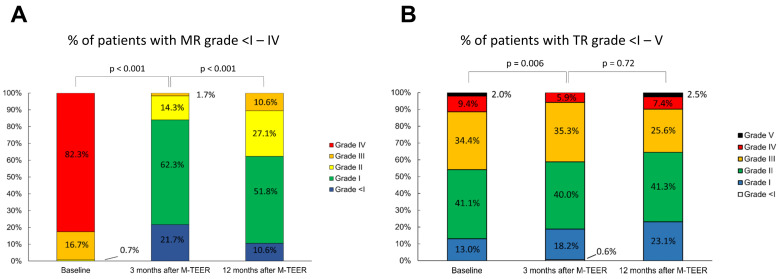
MR (**A**) and TR (**B**) grade at baseline, 3 months and 12 months after M-TEER. MR: mitral regurgitation; TR: tricuspid regurgitation, M-TEER: mitral valve transcatheter edge-to-edge repair.

Echocardiographic follow-up within 12 months after the procedure was available for 198 patients. MR reduction was sustained throughout the first three months after the procedure; however, residual MR grade increased significantly between the three month and 12 month follow-up (Figure 1A). TR improved by at least one grade in 60 patients (30.2%), while it did not improve in 138 patients (69.8%). Significant decrease in TR was seen after three months, with further reduction up until 12 months post procedure (Figure 1B).

### 3.2. Characteristics of Patients with and without TR Improvement

Patients with improved TR were more often female (63.3 vs. 41.3%, *p* = 0.04) and had lower body mass index (24.7 ± 4.1 vs. 26.3 ± 4.4 kg/m^2^, *p* = 0.01). Surprisingly, kidney function in patients with TR improvement was lower (estimated glomerular filtration rate (eGFR) 44.3 ± 15.7 vs. 50.2 ± 18.9 mL/min, *p* = 0.03) and NT-proBNP levels were considerably higher (7174.9 ± 9705.4 vs. 4732.5 ± 6219.9 pg/mL, *p* = 0.08). These patients had more severe TR at baseline (*p* = 0.006) and their right ventricular and right atrial dimensions trended to be smaller, although closely missing statistical significance (RAVI) 49.0 ± 22.7 vs. 55.1 ± 27.4 mL/m^2^, *p* = 0.11; basal RV diameter 46.9 ± 6.6 vs. 49.0 ± 8.3 mm, *p* = 0.06; mid RV diameter 32.0 ± 5.3 vs. 33.7 ± 6.8 mm, *p* = 0.07). MR grade IV at baseline was significantly more frequent in patients with postprocedural TR reduction (88.3 vs. 75.9%, *p* = 0.046). There was no relevant difference in residual MR severity at discharge; however, an absolute reduction in MR severity by three grades compared to baseline was achieved in significantly more patients with subsequent TR reduction (61.7 vs. 45.7%, *p* = 0.038). No relevant differences could be seen in left heart diameters, invasive hemodynamics, use of guideline-directed medical heart failure therapy or other comorbidities. Baseline characteristics are shown in Table 1. 

**Figure 2:**
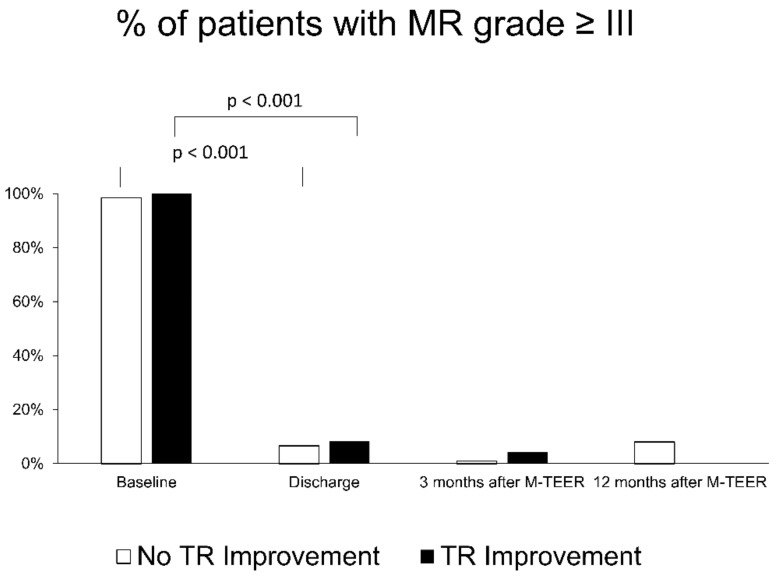
Course of MR grade in patients with (black columns) and without (white columns) secondary TR improvement after M-TEER. MR: mitral regurgitation; TR: tricuspid regurgitation, M-TEER: mitral valve transcatheter edge-to-edge repair.

Residual MR grade throughout 12 months was not significantly different between patients with improved and those with non-improved TR (Figure 2).

Logistic regression revealed female sex (odds ratio (OR) 2.997, 95% confidence interval (CI) 1.227–7.319, *p* = 0.016), baseline MR-Grade (OR 3.181, 95% CI 1.044–9.694, *p* = 0.042) and baseline TR-Grade (OR 2.653, 95% CI 1.488–4.728, *p* < 0.001) as factors independently associated with TR reduction (Table 2). 

### 3.3. Cardiac Remodeling in Patients with and without TR Improvement

Reduction in chamber volumes or diameters throughout 12 months occurred more frequently in patients with improved TR. A significantly larger decrease was seen in RAVI (delta −3.2 ± 18.2 vs. +3.9 ± 15.2 mL/m^2^ compared to baseline, *p* = 0.04) and left ventricular end-systolic diameter (LVESD) (delta −3.2 ± 7.6 vs. +1.9 ± 9.1 mm compared to baseline, *p* = 0.02). The diameter reduction of the RV base was also more pronounced, although just missing statistical significance (delta −0.6 ± 5.3 vs. +1.6 ± 5.7 mm, *p* = 0.06). Changes in volume and diameter are shown in Table 3. 

### 3.4. Twelve-Month Outcome in Patients with and without TR Improvement

**Figure 3:**
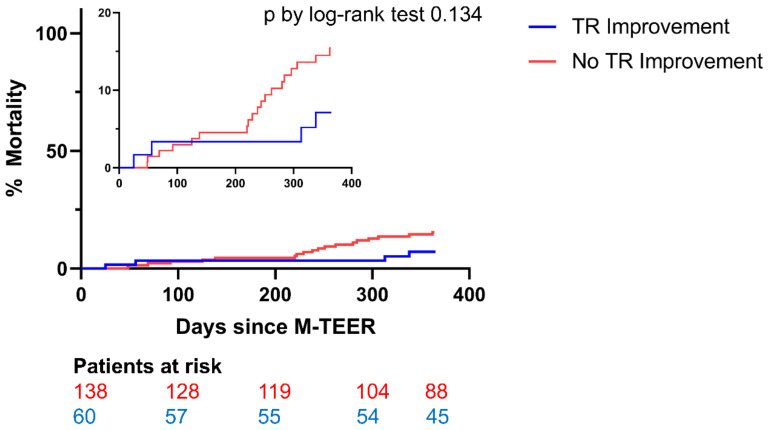
All-cause mortality in patients with (blue line) and without (red line) secondary TR improvement after M-TEER. MR: mitral regurgitation; TR: tricuspid regurgitation, M-TEER: mitral valve transcatheter edge-to-edge repair.

A total of 19 patients without TR improvement (13.8%) and 4 patients with TR improvement (6.7%) died within 12 months following M-TEER (*p* = 0.134). Kaplan–Meier plots began to diverge after 6 months (Figure 3). Due to the trend towards lower mortality in patients with regressed TR, multivariate Cox regression analysis was performed. TR improvement was independently associated with a reduction in risk of mortality (Hazard Ratio (HR) 0.333, 95% CI 0.112–0.996, *p* = 0.049) (Table 4).

## 4. Discussion

Concomitant TR is a frequent comorbidity in patients undergoing M-TEER, and its presence is linked to adverse outcomes following the procedure [3,4,5,6]. However, factors predicting TR improvement and thereby allowing for a better patient risk stratification are not well understood. This study evaluated factors predicting improvement in TR through mitral valve repair by TEER. 

The impact of concomitant TR on outcome after M-TEER is a matter of debate. Regression of TR after mitral valve repair has been reported by multiple studies [7,11,12,18]. Our study, among others, has now shown a significant positive effect of secondary TR regression on survival [7,11]. Interestingly, Adamo et al. reported that while baseline TR grade was not associated with adverse outcome, TR degree at short-term evaluation (median 79 days) after M-TEER is one of the strongest predictors of mortality [11].

Baseline characteristics of our cohort revealed more compromised organ function at baseline in patients with improved TR: lower LV-EF and kidney function, higher NT-proBNP levels, higher prevalence of MR grade IV and, most importantly, a higher grade of concomitant TR. However, RV function as assessed by tricuspid annular plane systolic excursion (TAPSE) was similar in both groups. Disagreeing with our data, Meijerink et al. described TR regression as mainly occurring in patients with RV dysfunction at baseline [19]. 

Importantly, we could not detect an impact of post-procedural residual MR grade on the course of TR as described in previous studies [11,12]. Notably, the number of patients with relevant residual MR (grade ≥ III) in our study was not sufficient to make reliable conjectures. However, baseline MR severity was independently associated with TR improvement after M-TEER. One might hypothesize that patients with higher MR grade at baseline benefit most from MR reduction and the consecutive decrease in volume overload. This volume decrease leads to reverse right heart remodeling and eventually results in a reduction of TR. A higher rate of TR regression in patients with significant TR at baseline has been shown by other studies [3,20]. Using logistic regression analysis, we could confirm that TR improves mainly when the relevant TR at baseline is present. TR most frequently develops secondary to abnormalities in left heart function, leading to pulmonary hypertension such as systolic or diastolic heart failure and valve disease [21]. Treatment of the underlying cause improves hemodynamic profiles, induces LV reverse remodeling and therefore commonly leads to a decrease in TR severity [9]. In their study, Toyama et al. found a reduction in sPAP after M-TEER to be independently associated with concomitant TR regression [20]. 

However, long-standing pulmonary hypertension can cause progressive RV dilation and failure. Accordingly, Kavsur et al. showed tricuspid annular dilation to hinder TR regression [12]. And Sordelli et al. identified pulmonary hypertension at follow-up as a significant positive predictor of TR progression after mitral valve surgery [22]. Even though we could not report on the course of pulmonary artery pressures after M-TEER, patients with a decrease in TR trended towards smaller RV diameters and right atrial volumes at baseline. These patients also showed a significant reduction in right heart chamber sizes at follow-up compared to baseline, while those with no improvement in TR did not. Based on our findings, the extent of baseline right heart remodeling seems to determine the extent of TR reduction after M-TEER. 

Strikingly, we found an independent association of female sex with TR regression after M-TEER. While, to our knowledge, no other study has linked gender to the course of TR after mitral valve repair, differences in myocardial remodeling between males and females have been described in animal studies, as well as in humans [23]. LV reverse remodeling has been found more frequently in females following M-TEER for functional MR [24,25]. However, specific data regarding RV function and TR are sparse [26]. Among other factors, lower rates of cell loss with preservation of cardiac function and dimensions in females have been discussed [23].

### Study Limitations

There are a number of limitations to our study that need to be addressed. Firstly, our data are generated by a monocentric and retrospective observational study. Secondly, echocardiographic grading of both MR and TR was carried out by experienced specialists at our center, independently from the procedure, but no independent core lab was involved. Furthermore, not all quantitative parameters for TR grading or exact measurements of tricuspid annular geometrics were available. Thirdly, we can only report a limited follow-up period of 12 months. However, as has been shown in numerous studies [7,11,12,18], relevant changes in TR severity occur in the first three months following M-TEER, a time period that is covered by our study.

## 5. Conclusions

One-third of patients undergoing M-TEER had a subsequent regression of concomitant TR. Severity of MR and TR at baseline, as well as female gender, were independently associated with TR regression. These patients also had smaller right ventricles and right atria and more pronounced right heart reverse remodeling compared to patients without TR improvement. A decrease in TR severity independently predicted mortality. These data further support a primarily conservative therapeutic approach regarding concomitant TR. However, echocardiographic follow-up for TR re-evaluation should be scheduled within three months following M-TEER, as non-improvement in TR may result in lower survival. Certain high-risk patients with TR due to advanced RV failure might benefit from early tricuspid-valve repair. However, further studies are needed to optimize treatment strategies.

## Figures and Tables

**Table 1:** Baseline characteristics of patients with and without TR improvement.

	Overall Cohort N = 198	No TR Improvement N = 138	TR Improvement N = 60	*p*
Age	77.7 ± 7.3	77.6 ± 7.2	78.7 ± 8.0	0.37
**Female Sex**	97 (49.0%)	**57 (41.3%)**	**38 (63.3%)**	**0.04**
**Body Mass Index (kg/m^2^)**	26.1 ± 4.7	**26.3 ± 4.4**	**24.7 ± 4.1**	**0.01**
Creatinine (µmol/L)	125.7 ± 67.2	127.4 ± 73.8	128.5 ± 55.3	0.9
**eGFR (mL/min)**	49.0 ± 18.4	**50.2 ± 18.9**	**44.3 ± 15.7**	**0.03**
Hemoglobine (g/dL)	12.6 ± 1.7	12.7 ± 1.7	12.5 ± 1.7	0.62
Troponin T (ng/L)	32.4 ± 26.1	32.2 ± 26.9	34.7 ± 26.1	0.55
NT-proBNP (pg/mL)	4273.4 ± 5274.8	4732.5 ± 6219.9	7174.9 ± 9705.4	0.08
Diabetes mellitus	52 (26.3%)	36 (26.1%)	17 (28.3%)	0.74
Coronary artery disease	118 (59.6%)	87 (63.0%)	32 (53.3%)	0.20
Obstructive lung disease	17 (8.6%)	13 (9.4%)	5 (8.3%)	0.81
Atrial fibrillation	118 (59.6%)	85 (61.6%)	34 (56.7%)	0.52
Baseline NYHA class				0.47
NYHA I	0	0	0	
NYHA II	39 (19.7%)	26 (18.9%)	13 (21.7%)	
NYHA III	127 (64.1%)	87 (63.5%)	37 (61.7%)	
NYHA IV	32 (16.2%)	24 (17.5%)	10 (16.7%)	
EuroSCORE II (%)	6.5 ± 6.0	6.6 ± 6.6	6.7 ± 4.9	0.89
STS-Score (%)	4.8 ± 4.7	5.0 ± 5.7	5.0 ± 3.2	0.87
LV-EF (%)	45.8 ± 15.7	47.0 ± 15.0	42.3 ± 16.7	0.08
V. cava inferior diameter (mm)	19.8 ± 5.9	20.4 ± 6.1	19.0 ± 6.1	0.22
TAPSE (mm)	19.7 ± 5.3	19.1 ± 5.3	19.3 ± 5.0	0.87
Baseline MR grade				0.12
Grade < I	0	0	0	
Grade I	0	0	0	
Grade II	2 (1.1%)	2 (1.5%)	0	
Grade III	38 (19.2%)	31 (22.6%)	7 (11.7%)	
Grade IV	157 (79.3%)	104 (75.9%)	53 (88.3%)	
MR grade at discharge				0.64
Grade < I	1 (0.5%)	1 (0.7%)	0	
Grade I	133 (67.2%)	89 (65.0%)	44 (73.3%)	
Grade II	49 (24.5%)	38 (27.7%)	11 (18.3%)	
Grade III	11 (5.6%)	7 (5.1%)	4 (6.7%)	
Grade IV	3 (1.5%)	2 (1.5%)	1 (1.7%)	
Absolute MR reduction by				
**3 grades**	**100 (50.5%)**	**63 (45.7%)**	**37 (61.7%)**	**0.04**
2 grades	78 (39.4%)	60 (43.5%)	18 (30.0%)	0.07
1 grade	17 (8.6%)	13 (9.4%)	4 (6.7%)	0.53
0 grades	3 (1.5%)	2 (1.5%)	1 (1.7%)	0.91
Functional MR	125 (63.1%)	84 (60.9%)	41 (68.3%)	0.32
LAVI (ml/m^2^)	70.5 ± 34.3	72.5 ± 33.6	65.1 ± 26.3	0.10
RAVI ml/m^2^	52.1 ± 26.5	55.1 ± 27.4	49.0 ± 22.7	0.11
LVEDD (mm)	58.4 ± 10.5	58.5 ± 10.4	58.3 ± 10.8	0.91
LVESD (mm)	42.5 ± 12.0	42.4 ± 11.7	44.4 ± 12.7	0.34
Basal RV diameter (mm)	48.1 ± 7.9	49.0 ± 8.3	46.9 ± 6.6	0.06
Baseline TR VC (mm)	7.3 ± 4.4	7.1 ± 4.4	8.2 ± 4.5	0.12
**Baseline TR Grade**				**0.006**
**Grade < I**	0	0	0	
**Grade I**	28 (14.1%)	**28 (20.3%)**	0	
**Grade II**	76 (38.4%)	**51 (37.0%)**	**25 (41.7%)**	
**Grade III**	73 (36.9%)	**46 (33.3%)**	**27 (45.0%)**	
**Grade IV**	18 (9.1%)	**11 (8.0%)**	**7 (11.7%)**	
**Grade V**	3 (1.5%)	**2 (1.4%)**	**1 (1.7%)**	
sPAP (mmHg)	48.5 ± 16.5	47.5 ± 15.0	50.5 ± 20.6	0.56
mPAP (mmHg)	31.7 ± 10.4	31.4 ± 10.6	33.4 ± 9.8	0.48
mPCWP (mmHg)	23.1 ± 7.7	22.9 ± 8.0	24.4 ± 7.2	0.53
mLAP (mmHg)	18.5 ± 5.9	18.6 ± 5.3	18.1 ± 7.2	0.76
LA v-wave (mmHg)	29.3 ± 12.8	29.5 ± 11.6	28.6 ± 15.6	0.80
mRAP (mmHg)	13.5 ± 6.0	13.0 ± 5.7	13.9 ± 6.3	0.47
PVR (dyn × s × cm^−5^)	268.4 ± 160.9	266.4 ± 164.7	290.7 ± 163.2	0.66
Cardiac Index (L/min/m^2^)	2.1 ± 0.8	2.0 ± 0.7	2.2 ± 1.0	0.41
Loop diuretics	165 (83.3%)	115 (83.3%)	50 (83.3%)	1.0
Betablocker	175 (88.4%)	122 (88.4%)	53 (88.3%)	0.99
ACE inhibitor/AT_1_ antagonist	143 (72.2%)	101 (73.2%)	42 (70.0%)	0.65
ARNI	27 (13.6%)	18 (13.0%)	9 (15.0%)	0.71
Aldosterone antagonist	107 (54.0%)	73 (52.9%)	34 (56.7%)	0.63
SGLT2 inhibitor	3 (1.5%)	2 (1.4%)	1 (1.7%)	0.92
Device Success	193 (97.5%)	134 (97.1%)	59 (98.3%)	0.61
Number of Devices implanted	1.7 ± 0.6	1.6 ± 0.6	1.7 ± 0.7	0.58
Mean transmitral gradient (mmHg) after M-TEER	3.8 ± 1.9	3.8 ± 1.9	3.8 ± 1.8	0.79
Hospital Stay (days)	7.3 ± 3.2	7.5 ± 3.4	7.2 ± 3.4	0.59
Need for CPR	0	0	0	1.0

TR: tricuspid regurgitation; eGFR: estimated glomerular filtration rate; NYHA: New York Heart Association; STS: Society of Thoracic Surgeons; LV-EF: left ventricular ejection fraction; TAPSE: tricuspid annular plane systolic excursion; MR: mitral regurgitation; LAVI: left atrial volume index; RAVI: right atrial volume index; LVEDD: left ventricular end-diastolic diameter; LVESD: left ventricular end-systolic diameter; RV: right ventricle; VC: vena contracta; sPAP: systolic pulmonary artery pressure; mPAP: mean pulmonary artery pressure; mPCWP: mean pulmonary capillary wedge pressure; mLAP: mean left atrial pressure; LA: left atrial; RAP: right atrial pressure; PVR: pulmonary vascular resistance; ACE: angiotensin converting enzyme; AT_1_: angiotensin receptor 1; ARNI: angiotensin receptor neprilysin inhibitor; SGLT-2: sodium-glucose linked transporter 2; M-TEER: mitral valve transcatheter edge-to-edge repair; CPR: cardiopulmonary resuscitation.

**Table 2:** Univariate and multivariate logistic regression for prediction or TR improvement within 12 months following M-TEER.

	Univariate Logistic Regression Analysis			Multivariate Logistic Regression Analysis		
	Odds Ratio	95% Confidence Interval	*p*	Odds Ratio	95% Confidence Interval	*p*
**Female Sex**	**2.455**	**1.314–4.585**	**0.005**	**2.997**	**1.227–7.319**	**0.016**
Body Mass Index	0.911	0.844–0.984	0.017	0.956	0.861–1.061	0.397
eGFR	0.982	0.965–0.999	0.038	0.987	0.964–1.011	0.280
NT-proBNP (per 1.000 pg/mL)	1.041	1.001–1.082	0.044	1.013	0.956–1.072	0.664
LV-EF	0.980	0.960–1.001	0.066	0.973	0.945–1.002	0.071
**Baseline** **MR-Grade**	**2.403**	**1.024–5.641**	**0.035**	**3.181**	**1.044–9.694**	**0.042**
LAVI	0.991	0.980–1.003	0.135	0.994	0.977–1.011	0.460
RAVI	0.990	0.978–1.003	0.139	0.988	0.964–1.013	0.360
Basal RV Diameter	0.965	0.927–1.005	0.086	0.953	0.885–1.025	0.197
**Baseline** **TR-Grade**	**1.664**	**1.168–2.371**	**0.005**	**2.653**	**1.488–4.728**	**<0.001**

TR: tricuspid regurgitation; M-TEER: mitral valve transcatheter edge-to-edge repair; eGFR: estimated glomerular filtration rate; LV-EF: left ventricular ejection fraction; MR: mitral regurgitation; LAVI: left atrial volume index; RAVI: right atrial volume index; RV: right ventricle.

**Table 3:** Evolution of heart chamber volume and diameter after 12 months, following M-TEER.

	No TR Improvement N = 138	TR Improvement N = 60	*p*
Delta LAVI (mL/m^2^)	1.0 ± 18.6	−0.6 ± 16.6	0.66
**Delta RAVI (mL/m^2^)**	**3.9 ± 15.2**	**−3.2 ± 18.2**	**0.04**
Delta LVEDD (mm)	−0.4 ± 7.9	−2.4 ± 7.8	0.30
**Delta LVESD (mm)**	**1.9 ± 9.1**	**−3.2 ± 7.6**	**0.02**
Delta Base RV (mm)	1.6 ± 5.7	−0.6 ± 5.3	0.06

M-TEER: mitral valve transcatheter edge-to-edge repair; TR: tricuspid regurgitation; LAVI: left atrial volume index; RAVI: right atrial volume index; LVEDD: left ventricular end-diastolic diameter; LVESD: left ventricular end-systolic diameter; RV: right ventricle.

**Table 4:** Univariate and Multivariate Cox Regression Analysis for prediction of mortality within 12 months following M-TEER.

	Univariate Regression Analysis			Multivariate Regression Analysis		
	Hazard Ratio	95% Confidence Interval	*p*	Hazard Ratio	95% Confidence Interval	*p*
Female Sex	0.985	0.435–2.233	0.971			
Body Mass Index	0.998	0.912–1.093	0.972			
eGFR	0.959	0.935–0.984	0.001	1.062	0.952–1.007	0.134
**NT-proBNP** **(per 1.000 pg/mL)**	**1.076**	**1.040–1.112**	**<0.001**	**1.062**	**1.017–1.110**	**0.007**
LV-EF	0.988	0.961–1.017	0.416			
Baseline MR-Grade	0.875	0.368–2.080	0.762			
LAVI	1.000	0.987–1.013	0.997			
RAVI	1.000	0.985–1.015	0.988			
Basal RV Diameter	1.034	0.983–1.087	0.200			
Baseline TR-Grade	0.878	0.555–1.388	0.567			
**TR-Improvement**	**0.449**	**0.153–1.319**	**0.145**	**0.333**	**0.112–0.996**	**0.049**

M-TEER: mitral valve transcatheter edge-to-edge repair; eGFR: estimated glomerular filtration rate; LV-EF: left ventricular ejection fraction; MR: mitral regurgitation; LAVI: left atrial volume index; RAVI: right atrial volume index; RV: right ventricle; TR: tricuspid regurgitation.

## Data Availability

Data available on request.
